# On Excessive Vomiting during Pregnancy

**Published:** 1855-11

**Authors:** 


On Excessive Vomiting During Pregnancy.—In an interesting thesis upon the non-coercible vomiting in pregnancy, and especially in relation to the induction of artificial delivery, M. Castaya has collected 58 cases that have been published by various authors. He distributes these, according to their mode of termination, into 5 groups:

1. Cases which proved fatal (24), without abortion occurring, and notwithstanding every variety of treatment.
2. Cases of recovery (11), after spontaneous abortion, or after the infant had died, although its expulsion was some time delayed.
3. Cases of recovery (3), without spontaneous abortion.
4. Fatal cases (3), notwithstanding spontaneous abortion; this occurring too late to be of avail, the patient's powers being exhausted.
5. Cases of artificial abortion (17). These are subdivided into two groups, successful (14), and unsuccessful (3). In 3 cases pregnancy was sufficiently advanced to admit of a living child being born, as well as of the mother's life being saved. Thus, in 58 cases, we have 30 deaths, and 28 recoveries. Of these last, 11 cases were due to spontaneous abortion, 3 to the medicines employed, and 14 to the operation.—(*Gaz. des Höp.* 1855. No. 85.) *London Lancet.*

